# Supplementary material for: Long-term monitoring of margays (Leopardus wiedii): Implications for understanding low detection rates
Source: PLoS One. 2021 Mar 1;16(3):e0247536. doi: 10.1371/journal.pone.0247536 (PMC7920358; doi:10.1371/journal.pone.0247536)
Supplement: Attachment — Submitted filename: Response to Reviewers_34073.odt [file pone.0247536.s004.odt]

Please ensure that your manuscript meets PLOS ONE's style requirements, including those for file naming. The PLOS ONE style templates can be found at
https://journals.plos.org/plosone/s/file?id=wjVg/PLOSOne_formatting_sample_main_body.pdf and
https://journals.plos.org/plosone/s/file?id=ba62/PLOSOne_formatting_sample_title_authors_affiliations.pdf
 
2. In your Methods section, please provide additional location information of the study site, including geographic coordinates for the data set if available.
We have provided the WSG84 lat long location from the centre of the study area (line 212)
3. In your Methods section, please provide additional information regarding the permits you obtained for the work. Please ensure you have included the full name of the authority that approved the field site access and, if no permits were required, a brief statement explaining why.
At the end of the first paragraph of the methods section we have stated that we got permission from the relevant authorities (Government of Belize and Belize Audubon Society). Lines 216-217.
4. Thank you for stating the following in the Acknowledgments Section of your manuscript:
"This research was funded by Panthera, 590 the Wildlife Conservation Society, the UK Natural
Environment Research Council, the Liz Claiborne Art Ortenberg Foundation, the North of
England Zoological Society, Brevard Zoo, Woodland Park Zoo, Virginia Tech, and the
Summerlee Foundation. The Government of Belize, Forest Department and Belize Audubon
Society provided invaluable logistical support."
We note that you have provided funding information that is not currently declared in your Funding Statement. However, funding information should not appear in the Acknowledgments section or other areas of your manuscript. We will only publish funding information present in the Funding Statement section of the online submission form.
We have removed this from the Acknowledgements section, and only included in the online form
Please remove any funding-related text from the manuscript and let us know how you would like to update your Funding Statement. Currently, your Funding Statement reads as follows:
"No: The funders had no role in study design, data collection and analysis, decision to publish, or preparation of the manuscript."
Please include your amended statements within your cover letter; we will change the online submission form on your behalf.

Thank you. We have updated our cover letter
5. We note that you have stated that you will provide repository information for your data at acceptance. Should your manuscript be accepted for publication, we will hold it until you provide the relevant accession numbers or DOIs necessary to access your data. If you wish to make changes to your Data Availability statement, please describe these changes in your cover letter and we will update your Data Availability statement to reflect the information you provide.

Thank you. We have updated our cover letter and new submission to reflect our Data Availability.
6. We note that Figure 1 in your submission contain map images which may be copyrighted. All PLOS content is published under the Creative Commons Attribution License (CC BY 4.0), which means that the manuscript, images, and Supporting Information files will be freely available online, and any third party is permitted to access, download, copy, distribute, and use these materials in any way, even commercially, with proper attribution. For these reasons, we cannot publish previously copyrighted maps or satellite images created using proprietary data, such as Google software (Google Maps, Street View, and Earth). For more information, see our copyright guidelines: http://journals.plos.org/plosone/s/licenses-and-copyright.

Figure 1 is similar to other Figures that we have published illustrating our long-term study site in Belize. We have now edited Figure 1 so that it is different than previous published versions. 
We require you to either (1) present written permission from the copyright holder to publish these figures specifically under the CC BY 4.0 license, or (2) remove the figures from your submission:
NA
7. Please include captions for your Supporting Information files at the end of your manuscript, and update any in-text citations to match accordingly. Please see our Supporting Information guidelines for more information: http://journals.plos.org/plosone/s/supporting-information.
We have included captions for the Supporting Information file 
All line indicated within the reviewer responses refer to the track-changed manuscript document!

# Reviewer 1
Reviewer #1: The authors studied the relative abundance of margays (Felis wiedii) along a 12-years period in a natural area in Belize. Their sampling design was established according to territory of big cats (jaguar and puma), by placing 20 stations with two camera-traps each and sampled for three consecutive months during the dry season. Authors claimed about the inconvenience of sampling small cats, like margays, with large grids with long interdistances between stations. In order to understand whether perceived populations can be affected by the sampling design, they included a line transect of camera-traps with low interdistance during two years and calculated some parameters to see their influence on estimated margay abundance.
I acknowledge the huge effort performed by authors, and the data presented could be remarkably interesting for the knowledge of a very rare species like the margay. However, I see several shortcomings on the way data were analyzed, by using simple linear correlations, T-tests, Chi2 tests. In my opinion, it is not a correct statistical approach. 
Several claims about detectability are raised along the paper, but authors do nothing to control for it. 
I guess that occupancy models will help to understand whether the sampling grid used for large cats can result in low detectability for margays, and this can correct naïve occupancy to actual occupancy. 
Thank you for raising the suggestion of occupancy modelling. It makes for an interesting analysis and discussion. If occupancy modelling is to be applied to camera trap data, it requires a large area to be sampled in order to ensure independence between camera sites while simultaneously sampling an adequate number of sites for parameter estimation. The interpretation of occupancy within camera trap studies is problematic, as camera locations are generally limited in number and not independently distributed with respect to the movement of individuals, especially for wide-ranging species. In our opinion, occupancy modelling is often inappropriately applied to camera trap data. Camera traps only detect species presence within an ~30 m2 zone in front of the camera trap. The interpretation of ‘occupancy’ thus becomes somewhat ambiguous, especially when used as a surrogate for abundance. To date, the peer review process has not questioned the use of occupancy models for data from single-season, small-scale camera survey grids, and its appropriate interpretation. We have now included a multi-season occupancy model and have discussed the outcomes. We use this analysis to demonstrate the high variability between years. With few margay detections per year, our top model estimates colonisation and extinction within the camera grid to fluctuate widely from one year to the next, resulting in derived estimates of occupancy which also fluctuate widely between the years. Across the 12 years, 19/20 locations were occupied by margays (naïve occupancy), which in light of the overall low detection is the most informative result. The multi-season analysis is thus useful in terms of indicating methodological limitations of their use for this species, at a minimum with this dataset. It strengthens our argument that the occupancy framework is unsuitable for revealing population structure from this dataset, corroborates our standing narrative and justifies our use of ‘simpler’ statistical methods. (see lines 280-311 and lines 421-477).

Moreover, the use of GLMMs would be necessary to account for several important thinks that are wrongly analyzed, like the influence of sampling interdistance on margays relative abundance. A single model including all relevant predictors (year, sex, competitors, etc), the interdistance as a covariate, and sampling station and individual as random factors, will yield more powerful information on the data. 

We thank the reviewer for the suggestions but a single GLMM incorporating multiple predictors and their interactions is not relevant for the questions that we are addressing, nor possible for use with our sparse dataset. Indeed, one of the main points of this manuscript is to illustrate what can (and cannot) be inferred from by-catch datasets, which are generally sparse. Our manuscript is about assessing rarity from sparse datasets. We are interested in abundance. Mark-recapture is the ideal framework for estimating abundance of an individually-recognisable species, like margays (see next comment for why we have not used this). However, as we point out in the manuscript, the camera data on small elusive tropical carnivores are generally too sparse for such an analysis. Thus, determining whether these species are rare or simply difficult to detect requires a different approach. Our aim was to show how a simple method could be used to distinguish between rarity and low detection rates. Thus, we present a method to assess rarity based on (1) the spatial characteristics of detections and (2) the response in detection rate (number of detections and number of individuals) to trapping effort. We have improved the narrative to make this clearer throughout. E.g. Lines 91-98, 111-140.

This incredible long sampling scheme will be adequate to capture-recapture models for demography of the margays.
The data are not adequate for abundance estimation by capture-recapture methods. Robust mark-recapture analysis requires an adequate sample size of recaptures (per year or across years). In this study, recapture rate per individual was low, with most individuals detected once each, insufficient for robust abundance estimation via capture-recapture models. Lines 115-121
Furthermore, I think that the text is very difficult to read fluidly, and several paragraphs showed statements that are very subjective and not supported by any reference.
We have improved the narrative throughout.
I guess that the article needs to be re-analyzed by using more adequate statistical approach.
For margay, the current literature makes relatively bold statements on the species status, with rarity as the general framework. Our extensive search of the published literature for camera trap studies on margays shows low sample sizes with infrequent recaptures. Our dataset adds to this understanding. We have thoroughly addressed the limitations of our dataset with respect to the statistical approaches suggested by the reviewer. The sparsity of our dataset is representative of datasets of small tropical carnivores like margays, while having the advantage of 12 years of data. Appropriate analyses for such data are relatively simple. It is unfortunate that publishing about these species is stalled by the popular demand for sophisticated models that cannot be fit to such sparse data. Our approach is useful and informative because it indicates how detections were distributed between individuals through space and time and how this may be interpreted in terms of local population status. We have improved the narrative to clarify this and included occupancy modelling to illustrate the difficulty of interpreting this statistical approach when applied to such data. (see Lines 91-98, 111-140, 280-311, 421-477)

Line 41: What means micro-placement? Please clarify
We have changed the word to micro habitat and improved the sentence for clarity. Lines 40-43, 45, 48)
Line 43: there will be also few captures
This is not necessarily true. Cameras placed optimally at the microhabitat level, but widely spaced across the landscape can result in many detections of the same individual at the same camera location. However, the number of spatial recaptures (same individual at different locations) will be low because they do not travel far enough to reach neighbouring camera locations. 

Line 44: What means micro-level? Please clarify
We have changed this to micro habitat level in response to the earlier comment. Line 48
Line 154: What means pre, post, please be clearer. It is problematic to gather stations with different interdistance (2000, 700, 140 m) to the original design. During the last three years interdistance was a third of the original design. Justify
We have improved the narrative, indicating that we deployed extra cameras at different times of the year in addition to the dry season survey which comprised 20 camera stations at fixed locations. The shorter mean distance in the latter three years is due to the additional cameras deployed at higher density within these years (see Table 1). However, the mean distance between neighbouring cameras in the 20-station dry season surveys remained consistent every year at 2.0 km, as indicated in the Methods. Lines 236-238.
Line 209. If you are not confident about the role that the capture probability has on the data, why do not applying occupancy models accounting for imperfect detectability? I guess that your data are adequate for this approach by using the 20-stations design.
Please see our previous response about the use of occupancy modelling with this dataset. Occupancy modelling uses detection probability to estimate occupancy. Low detection probability is problematic for both mark-recapture and occupancy modelling, resulting in large confidence intervals in parameter estimation, and unstable model choice. We have now included an occupancy model which demonstrates that it is difficult to interpret the outcome from by-catch data within an ecological framework. Lines 280-311, 421-477
Line 218. This can be true for widely spaced stations, but what happens when stations are too close like in the last three years?
We have improved the narrative to make it clearer what spatial recaptures means in relation to recaptures. 
Line 220. This paragraph is confuse. Try to use the two concepts (spatial vs temporal) recaptures along the paper. I guess that recaptures are a combination of both spatial and temporal recaptures.
We have improved the narrative to clarify this. We defined a ‘spatial recapture’ as detection of the same individual at > 1 location. As an individual is unable to occupy different locations at the same time, this is both spatial and temporal. A ‘recapture’ simply means detected again, regardless of whether that it is at the same or different location. Lines 317, 335-339.
Line 236: It is very difficult to follow this complex way of using your dataset, by adding/deleting data at your convenience.
We have improved the narrative explaining the reason for using these three well-defined datasets. Lines 333-339.
Line 277: Occupancy models will yield information about survival, mortality, etc.
Occupancy modelling does not consider individuals and thus does not measure individual survival. As indicated, the majority of individuals in our dataset were detected once only, unsuitable for such analyses. Likewise, with the majority of individuals detected only once, we are unable to analyse the data within an open population mark-recapture framework necessary for estimating survival or mortality. However, we do assess longevity within the limitation of our dataset.

Line 284: “Most detections on the forest floor occurred at night”, but, have you detected the species at other forest levels? If not, you can delete it.

We have removed “on the forest floor”. Lines 410.
Line 285; Is this overlap high? Please, use some kind of reference to the values showed.
The Methods section indicates that this is a measure ranging between 0-1. A value of 0 indicates no overlap and a value of 1 indicates identical activity patterns. Lines 275-278.
Line 322: Using simple linear correlations for such a data is a poor statistical approach. There are several uncontrolled variables that, maybe, can be interfering with the observed pattern.
At the large-scale, the habitat of our camera grid is homogenous (dense lowland tropical forest). We recognise that there is noise in this dataset associated with the multitude of unmeasured micro-habitat differences around each camera location, for example undergrowth type and density, canopy height, density and proximity of alternative trails, proximity of waterways etc. By using a large number of camera locations (N = 250) we minimise the effects of this noise. Our interest is in effect of trap effort, which we are able to detect despite the noise associated with other unmeasured variables. We acknowledge that if these other variables were known and accounted for then the model fit may improve further and provide additional information.
The statistical approach used is rather dull, when there are very flexible and powerful tools easy available like GLMMs that would be suitable for such a heterogeneous data set. Using the number of independent detections by individual/year as the response variable, you can model several responses to grid design, year, sex, competitors, recaptures, and so on, while controlling for the spatial situation of stations (random effects), the identity of individuals (random effects), and the sampling effort. In a similar way, actual occupancy can be examined controlling for imperfect detection by application of occupancy models and assessing the probabilities of local colonization and extinction along the study period. Individuals’ capture histories surely will yield some interesting data about survival and other demographic parameters. And even, interesting information about density.
Whether or not a statistical approach is dull is highly subjective and irrelevant. We used the methods that were most suitable for a dataset which consists of a large number of individuals but has sparse data points for each individual. Please review the data distribution in more detail (Table 2). The majority of individuals were detected only once during the 12-year study thus it is not appropriate to test for variation between individuals in detection rate with any number of possible independent variables. 
There are important reasons for rarity of margays in the study area: the competence with ocelots, and the arboreality, which means less detectability in stations placed on the forest floor.
We do not conclude rarity. Based on the patterns we observe, we question the frequent conclusion that a species is rare because it has few captures. Regarding competition with ocelots, Oliveira et al. 2010 found an inverse relationship between the density of ocelots and smaller cats, however no data were reported on the sample sizes used for density estimation, and to our knowledge, subsequent studies have not found a similar relationship. 
# Reviewer 2
Reviewer #2: This manuscript reflects an exceptional effort to improve our knowledge on a species that is difficult to study. It is commendable to see a publication of a very long study effort, which showed the benefit of long-term research on a seemingly rare animal. Apart from several minor grammatical pick-ups (please see below), I would only have a few suggestions that may improve this publication.
1) The authors should reflect more on the ecology and life history of margays. What is known about their habitat selection (e.g. is this study site thought to be a representative habitat of where these cats would thrive, given the background of the site – Line 129. This of course would have implications on the density of these animals in the area)? Similarly, what is the life history of margays – expected longevity (is there anything known about captive populations for instance, or other similar felids), reproductive output; and how does this link with the results obtained?
We have added to the Introduction narrative about the ecology and life history of margays. Lines 74-83.
2) How does the high density of other felids in the area (pumas, ocelots, and jaguars) (Line 135) impact on margays? Is there interspecific competition for prey, predator- prey interactions, other interspecific competition, that is expected to impact on margays? If the author’s expectation is different to the observation, please provide some suggestions of why you may not have observed the expected outcomes? The manuscript briefly touched on this in the discussion, but the “ocelot effect” was not observed (despite the high ocelot density), and the explanation for this was not explored/provided. Similarly, for instance, smaller home ranges observed (Lines 510-513) could be a reflection of inter- and intra- specific competition, rather than just camera placement?
We have added discussion of interspecific competition and predation in the Discussion; and suggested why the ocelot effect was not observed in the study area. As there are numerous carnivores and omnivores co-existing with margays in this study area (grey foxes, jaguarundi, ocelots, tayras, coatis, snakes) which potentially hunt the same prey. In the absence of diet studies on margays and these other species in the study area, we cannot comment on interspecific competition for prey. Lines 703-712.
3) Finally a small improvement on the conclusions could be to reflect on suggested optimal time the cameras should be deployed for (i.e. what does a ‘long-term study’ mean – would 2-3 years be sufficient? 12 years is possibly an unrealistic expectation, and/or other improvements should be included). If these cats are semi-arboreal, is there a chance of changing the placement of the cameras and using them in canopy (at what stratification would you expect margays?). In addition, can scat analyses, tracks and other markings be of use?
Any long-term study should reflect the longevity of the target species. We have now recommended that where long-term studies are not feasible that survey periods be extended for as long as possible within the temporal limits for a closed population. We discuss options for improving camera deployment to increase detectability, including the placing cameras in the canopy. Scats and tracks can be used for detecting species presence (not useful for population assessment); however, our earlier work from the same study area showed that detection rates of margay tracks (Harmsen et al. 2010) and scats (unpubl data) were extremely low so we do not recommend that as a method here. Lines 746-753, 785-789.

Harmsen BJ, Foster RJ, Silver S, Ostro L, Doncaster CP. Differential use of trails by forest mammals and the implications for camera-trap studies: A case study from Belize. Biotropica. 2010;42: 126–133. doi:10.1111/j.1744-7429.2009.00544.x
4) The authors have suggested that Animal Ethics approval was not applicable to this study? Why was this not required for a camera trap study?
Animal Ethics approval is not required for the use of camera traps. Camera trapping is a widely-accepted, non-invasive method of monitoring wild species.  
Lines 50-52 – examples are convoluted – consider revising sentence.
We have revised the sentence. Lines 51-56.
Line 86 – parenthesis “(“ can be deleted.
Changed to : as it concerns a list. Line 105
Line 103 – missing full-stop after the word design.
Section changed. Lines 141-151.
Line 139 vs 142 state that 19 vs 20 locations were surveyed for 12 seasons
We have changed to 20 locations in the Figure legend. Line 220.
Line 154 put a space after pre-,
Deleted in the changed narrative. Lines 236-239.
Lines 168 -171 are meant to go above the table (same for other table captions)
We followed the PLoSONE guidelines for tables which state  - indicating the table title at the top and legend text and footnotes at the bottom. 
Line 207 locations-years should be location-years
Changed. Line 324.
Line 237 and 240 and 242 put space before km (check throughout the manuscript). 
Included. Lines 358, 362, 365.
Line 330 need a space before .
Included. Line 507.
Line 333 need a space after 0.0044
Included. Line 509
Line 336 need a space after 0.53
Included. Line 513
Line 156, 452, 527, 528 need space between number and m (check throughout the manuscript)
We have corrected throughout. Lines 240, 629, 680, 727
Line 480 need a parenthesis “)” after [13]
Included. Line 657. 
Line 494 clarify why/how your results are suggestive of a stable population
We have removed this statement as we do not have evidence that the population size is stable through time. Line 672.
Line 502 missing full stop
Included. Line 680.
Line 510 missing parenthesis “)”
Parenthesis included. Line 689
Line 513, 534. 543, 553 references are spelled out
Changed and updated. Lines 691-692, 734, 746, 761.
Line 540 14 years or 12 years?
The data were collected across 14 years (2003 until 2017), however no data were collected in 2009 or 2010. Therefore, the data were collected in 12 different years. Lines 223-227.
Line 566 change [] to ()
Changed. Lines 775-776

# Reviewer 3
Reviewer #3: The manuscript is well organized and compiles 12 years of research in detailed analyses, regarding detectability. I made few comments on some parts of the text, listed at the end of this concept.

I just have a couple of comments regarding the discussion section. As mentioned by the authors, the study site at Cockscomb Basin Wildlife Sanctuary, represents a pristine area with overall good conservation conditions. To what extend it is safe to extrapolate data from a pristine area like that, to other study sites likely to hold a different quality of habitats for margays? This is of particular interest, since some instances of the inferences made by the authors refer to the conservation status assigned by the IUCN to the species.
We agree that Cockscomb represents a pristine area which may not be representative of many areas where margays exist; we have indicated this specifically to clarify this. Lines 700-702.
A paragraph talking about the implications of habitat quality on felid species behavior and its effect on results obtained from camara trap assessments would be great.
As our study does not investigate the effect of variation in habitat quality, we do not feel well-placed to discuss its influence on cat behaviour. However, we do discuss how camera trapping could be modified to improve detection rates of margay. Lines 746-753.
On the other hand, there is little discussion on the differences in number of detections throughout the years at the study site. I am pretty sure, that based on their knowledge of the study area, the authors will be able to add some information on other environmental conditions that could potentially drive this changes in detections (i. e. climate variability, specially, El Niño event that was particularly strong in 2015-2016, and La Niña event, that reported a strong effect between 2010-2011, these two lapses yielded low values).
We agree with the reviewer that such events could have considerable impact on rodent populations and thus impact rodent predators, such as margay. We have added a multi-season occupancy model to estimate detectability (probability of detection if present) and occupancy (derived from colonisation and extinction probabilities) for every year of the study. The best-fitting model suggests that ‘occupancy’ of the camera grid fluctuates widely between the years (like our raw detection rates), while detectability remains constant. We infer that these fluctuations reflect the fact that the survey design was sub-optimal, with cameras placed on wide trails not often frequented by margays (they are by-catch species). Thus, margay detections by cameras were rare, sporadic events. Furthermore the dataset is too sparse to draw conclusions about environmental drivers, with a sample size of only one El Nino and El Nina event during the study. Lines 421-477.

Taking into consideration the quality and robustness of the information presented, I consider the manuscript to be published with minor additions and changes.
SUGGESTIONS
Lines 56 and 57: please review closing parenthesis, starting at: “(e.g. jaguars (Panthera onca): …”.
Included. Line 62
Lines 64 and 65: please reorganize the sentence: “we assess population status of a neotropical, semi-arboreal felid, the margay (Leopardus wiedii), …” Suggestion: we assess population status of the margay (Leopardus wiedii),a neotropical, semi-arboreal felid,…
We have reorganised and changed the sentences. Lines 71-72, 78-79.
Line 68: include “As mentioned” in: Margays are small, semi-arboreal felids weighing 2 to 4 kg,…”; suggestion: As mentioned, margays are small, semi-arboreal felids weighing 2 to 4 kg,…
We have reorganised and changed the sentences. Lines 71-72, 78-79.
Lines 70 to 72: sounds a little contradictory, that the authors affirm: “The geographic ranges of both species almost completely overlap, making margays potential by-catch in camera trap survey grids designed for jaguars [13,14].” which is just the point of controversy of this particular study. It would be more coherent, in the context of the logic of the paper, for the authors to previously introduce a sentence with the following statement: It has been assumed that species that overlap in their geographic ranges and are considered sympatric in some areas can be effectively detected under single experimental designs, that not necessarily take into consideration behavioral microhabitat preferences.
We have changed the text as suggested. Lines 59-64
Lines 92 to 94: We address this by presenting a simple method to assess rarity based on the spatial characteristics of a population and trapping effort, and apply it to the margay ‘by-catch’ data obtained from a long-term jaguar-monitoring program. The authors use the same heading of this sentence in line 111: “Here, we use a simple method to make inferences about the population status of margays…” it would sounds better if the authors say: Here we used the above mentioned approach to make inferences about the population status of margays…
We have rewritten this section. Lines 111-121.
Lines 129 to 130: It is important to provide a geographic context associated with the study site, suggestion: “The study was conducted in the moist broad-leaved tropical forest of the Cockscomb Basin Wildlife Sanctuary (from hereon, CBWS or sanctuary), a natural area located in the south-central zone of Belize created to protect the forests, the fauna and the hydrographic basins of approximately 400 square kilometers of the eastern slopes of the Maya Mountains…
Suggestion included. Lines 207-209.
Line 502: include a period at the end in: “cameras spaced 200 – 500m apart [12], Vanderhoff pers comm).
Included. Line 680.
